# Validation of the Barcelona-MRI predictive model when PI-RADS v2.1 is used with transperineal prostate biopsies

**DOI:** 10.1590/S1677-5538.IBJU.2024.0204

**Published:** 2024-07-20

**Authors:** Juan Morote, Nahuel Paesano, Natàlia Picola, Jesús Muñoz-Rodriguez, Xavier Ruiz-Plazas, Marta V. Muñoz-Rivero, Ana Celma, Gemma García-de Manuel, Berta Miró, Pol Servian, José M. Abascal

**Affiliations:** 1 Hospital Univeritari Vall d'Hebron Department of Urology Barcelona Spain Department of Urology, Hospital Univeritari Vall d'Hebron, Barcelona, Spain; 2 Universitat Autònoma de Barcelona Department of Surgery Bellaterra Spain Department of Surgery, Universitat Autònoma de Barcelona, Bellaterra, Spain; 3 Clínica Creu Blanca Barcelona Spain Clínica Creu Blanca, Barcelona, Spain; 4 Hospital Universitari de Bellvitge Department of Urology Spain Department of Urology, Hospital Universitari de Bellvitge, Hospitalet de Llobregat, Spain; 5 Hospital Universitari Parc Tauli Department of Urology Sabadell Spain Department of Urology, Hospital Universitari Parc Tauli, Sabadell, Spain; 6 Hospital Universitari Joan XXIII Department of Urology Tarragona Spain Department of Urology, Hospital Universitari Joan XXIII, Tarragona, Spain; 7 Hospital Universitari Arnau de Vilanova Department of Urology Lleida Spain Department of Urology, Hospital Universitari Arnau de Vilanova, Lleida, Spain; 8 Hospital Universitari Josep Trueta Department of Urology Girona Spain Department of Urology, Hospital Universitari Josep Trueta, Girona, Spain; 9 Vall d'Hebron Research Institute Statistic Unit Barcelona Spain Statistic Unit, Vall d'Hebron Research Institute, Barcelona, Spain; 10 Hospital Univeritari Germans Trias i Pujol Department of Urology Badalona Spain Department of Urology, Hospital Univeritari Germans Trias i Pujol, Badalona, Spain; 11 Parc de Salut Mar Department of Urology Barcelona Spain Department of Urology, Parc de Salut Mar, Barcelona Spain; 12 Universitat Pompeu Fabra Department of Medicine and Health Sciences Barcelona Spain Department of Medicine and Health Sciences, Universitat Pompeu Fabra, Barcelona, Spain

**Keywords:** Prostatic Neoplasms, Magnetic Resonance Imaging, Diagnosis

## Abstract

**Purpose:**

To validate the Barcelona magnetic resonance imaging predictive model (BCN-MRI PM) in men with pre-biopsy multiparametric MRI (mpMRI) reported with the Prostate Imaging Reporting and Data System (PI-RADS) v2.1, followed by transrectal and transperineal prostate biopsies.

**Materials and Methods:**

Prospective analysis of 3,264 men with PSA >3.0 ng/mL and/or abnormal digital rectal examination who were referred to ten participant centers in the csPCa early detection program of Catalonia (Spain), between 2021 and 2023. MpMRI was reported with the PI-RADS v2.1, and 2- to 4-core MRI-transrectal ultrasound (TRUS) fusion-targeted biopsy of suspected lesions and/or 12-core systematic biopsy were conducted. 2,295 (70.3%) individuals were referred to six centers for transrectal prostate biopsies, while 969 (39.7%) were referred to four centers for transperineal prostate biopsies. CsPCa was classified whenever the International Society of Urologic Pathology grade group was 2 or higher.

**Results:**

CsPCa was detected in 41% of transrectal prostate biopsies and in 45.9% of transperineal prostate biopsies (p <0.016). Both BCN-MRI PM calibration curves were within the ideal correlation between predicted and observed csPCa. Areas under the curve and 95% confidence intervals were 0.847 (0.830-0.857) and 0.830 (0.823-0.855), respectively (p = 0.346). Specificities corresponding to 95% sensitivity were 37.6 and 36.8%, respectively (p = 0.387). The Net benefit of the BCN-MRI PM was similar with both biopsy methods.

**Conclusions:**

The BCN-MRI PM has been successfully validated when mpMRI was reported with the PI-RADS v2.1 and prostate biopsies were conducted via the transrectal and transperineal route.

## INTRODUCTION

Risk-stratified prostate cancer (PCa) screening, based on serum prostate-specific antigen (PSA) and magnetic resonance imaging (MRI), is currently recommended by the European Union (1). The new paradigm for PCa screening is focused on the early detection of clinically significant PCa (csPCa) (2). This paradigm change is based on evidence reported by the European Randomized Screening Prostate Cancer study in 2009. In this randomized trial, the hazard ratio for cause-specific death in the screening arm, and its 95% confidence interval, as compared with the control arm, reached a significance of 0.80 (0.65 to 0.98) at 8.8 years of follow up (3). This significant reduction of PCa-specific mortality has been maintained after 22 years of follow up in the Göteborg Randomized Population-Based Prostate Cancer Screening Trial (4). The European Association of Urology currently proposes the use of risk-stratified pathways, based on predictive models, for improving csPCa screening by reducing the demand for MRI exams, maximizing the detection of csPCa, and decreasing unnecessary prostate biopsies and over-detection of insignificant PCa (iPCa) (5, 6).

The Barcelona-MRI predictive model (BCN-MRI PM) for individualizing the risk of csPCa detection in prostate biopsies was developed due to the absence of csPCa risk calculators using the Prostate Imaging Reporting and Data System (PI-RADS) v2.0, and six other independent clinical predictive variables without range limitations, namely: age (years), PCa family history (no vs. yes), type of prostate biopsy (initial vs. repeated), serum PSA (ng/mL), digital rectal examination (DRE: normal vs. suspicious), MRI-derived prostate volume (mL), and PI-RADS score from 1 to 5 (7). The BCN-MRI PM development cohort included 1,486 men, with serum PSA >3.0 ng/mL and/or suspicious DRE, who underwent pre-biopsy multiparametric MRI (mpMRI) reported with PI-RADS v.2.0, followed by 2- to 4-core MRI-transrectal ultrasound (TRUS) fusion-targeted biopsy of PI-RADS >3 lesions and 12-core systematic biopsy, but only a 12-core systematic biopsy in those negative MRI (PI-RADS 1 or 2). This development cohort was prospectively recruited in a single academic institution between 2016 and 2019. Additionally, an external validation was conducted in 946 men, who underwent the same PCa suspicion criteria and diagnostic approach as those in the development cohort, in two centers from the Barcelona metropolitan area within the same period (8). The BCN-MRI risk calculator was designed for the easy and quick assessment of individual risk of csPCa, with the novelty of selecting the appropriate threshold for prostate biopsy decision, free available without cost at the https://mripcaprediction.shinyapps.io/MRIPCaPrediction/ (accessed on March 29, 2024). The BCN-MRI PM has been compared with the prestigious Rotterdam-MRI PM in a head-to-head analysis conducted in the external validation cohort. A better overall performance of the BCN-MRI PM was observed, especially in men with PI-RADS of 3 and 4. Additionally, it was observed that 22% of men included in this analysis presented age, serum PSA, or prostate volume out of the range accepted by the Rotterdam-MRI risk calculator (9).

Current predictive models require validation in populations where they are intended to be applied, even if the event of changes in the characteristics of the population from which the development cohort came or changes in the diagnostic procedure. These validations are necessary to ensure the ongoing accuracy of individual predictions (10).

Two relevant changes have recently been incorporated into the early diagnostic approach to csPCa. First, the PI-RADS v2.1 is currently followed for reporting MRI findings, and second, transperineal route for prostate biopsies is suggested for avoiding the infectious complications of prostate biopsies (11, 12). We hypothesise the BCN-MRI predictive model will be successfully validated in when PI-RADS v2.1 is employed for reporting pre-biopsy MRI, and prostate biopsies conducted even by transrectal and transperineal route. The present study aims to validate the BCN-MRI PM in a csPCa opportunistic screening program where the diagnostic approach employed the PI-RADS v2.1 for reporting MRI, and transperineal or transrectal prostate biopsies.

## MATERIAL AND METHODS

### Design, setting, and participants

This is a prospective study conducted in 3,264 men with the inclusion criteria of (i) suspicion of PCa based on serum PSA of >3.0 ng/mL and/or suspicious DRE, (ii) pre-biopsy mpMRI reported with PI-RADS v2.1, and (iii) prostate biopsy following the scheme of 2- to 4-core MRI-transrectal ultrasound (TRUS) fusion-targeted biopsies and 12-core systematic biopsy in men with PI-RADS ≥ 3, but only a 12-core systematic biopsy in those with PI-RADS <3. This trial was conducted in ten centers participating in the csPCa early detection program of Catalonia (Spain), a region with 7.9 million inhabitants, between January 1, 2020, and June 30, 2023. Reported cases were consecutive in each participant center. A subset of 2,295 men (70.3%) underwent prostate biopsy in six participant centers where transrectal prostate biopsy was exclusively employed, while 969 (29.7%) underwent biopsies in three other centers exclusively employing transperineal biopsies. The exclusion criteria were men with previous diagnosis of PCa, multifocal high-grade prostatic intraepithelial neoplasia, and atypical small acinar proliferation. Men recruited in one participant center where transperineal prostate biopsies followed a mapping scheme for targeted biopsies were not included in this analysis. This project was approved by the ethics committee of the coordinating center (PRAG02/2020), with participants signing an informed consent.

### CsPCa suspicion and diagnostic approach

PCa suspicion was based mostly on a serum PSA >3.0 ng/mL, while 95 men (2.9%) exhibited a suspicious DRE with a serum PSA of 3.0 or lower. Men suspected of having PCa were referred to the nearest participating center of the csPCa early detection program. MpMRI was conducted at each participant center using a 1.5 or 3 Tesla scan with a pelvic phased-array surface coil. The acquisition protocol included T2-weighted imaging (T2W), diffusion-weighted imaging (DWI), and dynamic contrast-enhanced (DCE) imaging, according to the guidelines of the European Society of Urogenital Radiology (13). MpMRI exams were reeded by local expert radiologists reporting with the PI-RADS v2.1 (11). All prostate biopsies were performed using freehand technique and software MRI-TRUS fusion image for targeted biopsies in 42.8%, while cognitive fusion was employed in 67.8%. Uropathologists examined the biopsy material in each pathology department and reported PCa using the International Society of Urologic Pathology grade group (GG) classification. CsPCa was considered when the GG was 2 or higher (14).

## Statistical Analysis

Statistical analysis was conducted after harmonization of anonymized datasets. The data were prospectively collected and reported according to the Standards of Reporting for MRI-targeted Biopsy Studies (START) to describe the study population (15). Quantitative variables are described using medians and interquartile ranges (25th–75th percentiles), while qualitative variables are described using numbers and percentages. Quantitative variables were compared between groups using the Mann–Whitney U test. Qualitative variables were compared between groups using Pearson's chi-square test. Relative risk (RR) of csPCa and 95% confidence intervals (CI) were assessed. Calibration of the BCN-MRI PM was conducted for both prostate biopsy routes. Discrimination of csPCa from the BCN-MRI PM in each prostate biopsy group was analyzed with receiver operating characteristic (ROC) curves, and the areas under the curve (AUC) were compared with the DeLong test. Specificities corresponding to selected sensitivities with clinical interest were compared and avoided prostate biopsies and loss of csPCa estimated. Net benefit of the BCN-MRI PM over biopsy all men was evaluated through decision curve analysis (DCAs). A p value of <0.05 was considered statistically significant. The data were analyzed using the Statistical Package for the Social Sciences (version 29.0; IBM Corp., Armonk, NY, USA).

## RESULTS

Baseline characteristics of both subsets of men who underwent transrectal or transperineal route for prostate biopsy are summarized in Table-1. We note that baseline characteristics were similar in both subsets. The median interval from MRI exam to prostate biopsy was 27 days. However, csPCa was detected in 940 men (41%) who underwent transrectal prostate biopsy, and in 445 men (45.9%) who underwent transperineal prostate biopsy (p =0.016). The rates of iPCa detection were 16.3% and 17.4%, respectively (p =0.441).

**Table 1:** Characteristics of the study cohort according to the utilized prostate biopsy route.

Characteristic		Route of prostate biopsy		p Value
		Transrectal	Transperineal	
Number of men, n (%)		2,295 (70.3)	969 (29.7)	-
Median age, years (IQR)		68 (62-73)	68 (62-74)	0.556
Median serum PSA, ng/mL (IQR)		7.1 (5.2-11)	7.4 (5.4-10.7)	0.294
Abnormal DRE, n (%)		132 (25.7)	68 (26.0)	0.214
Median prostate volume, mL (IQR)		55 (40-79)	54 (39-76)	0.178
Prior negative prostate biopsy, n (%)		690 (30.1)	273 (28.1)	0.294
Family history of PCa, n (%)		121 (5.3%)	67 (6.9)	0.231
**PI-RADS, n (%)**				
	1-2	230 (10.0)	83 (8.6)	0.427
	3	565 (24.6)	200 (20.6)	0.235
	4	987 (43.0)	459 (47.4)	0.189
	5	513 (22.4)	227 (23.4)	0.201
**Overall PCa detection, n (%)**		1,315 (57.3)	651 (67.2)	0.001
	csPCa, n (%)	940 (41.0)	445 (45.9)	0.016
	iPCa, n (%)	375 (16.3)	169 (17.2)	0.441

IQR = interquartile range; n = number; PI-RADS = prostate imaging-reporting and data system; PCa = prostate cancer; csPCa = clinically significant PCa; iPCa = insignificant PCa.

**Figure 1:**
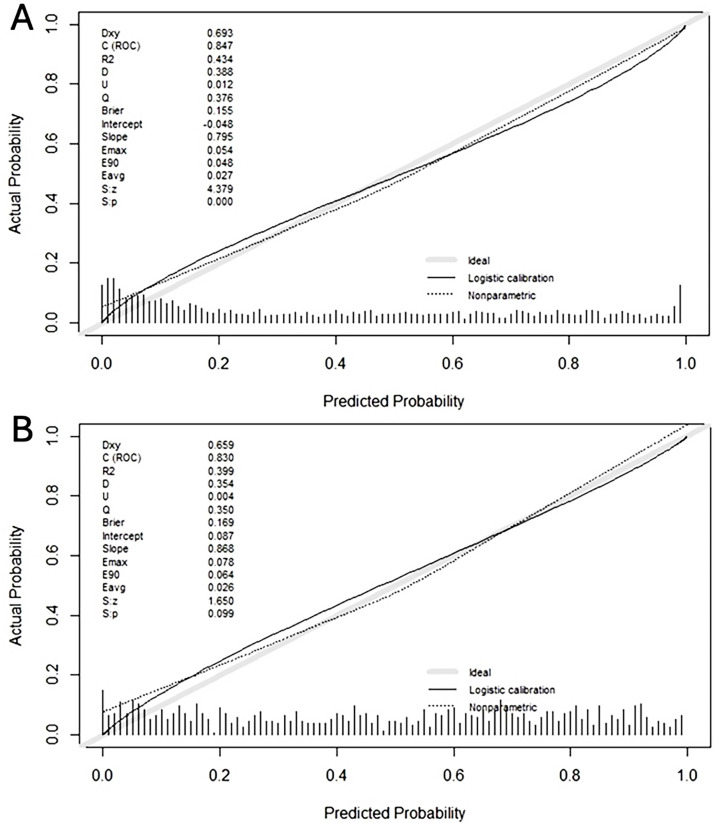
Calibration curves of the BCN-MRI PM in transrectal prostate biopsies (A) and transperineal prostate biopsies (B).

Calibration of the BCN-MRI PM in both subsets was very good. Similar calibration curves between predicted risks and observed csPCa cases in transrectal and transperineal prostate biopsies were observed. Both calibration curves showed a small over-estimation in lowest predictive probabilities of csPCa and minimal under-estimation in the higher predictive probabilities of csPCa, but both were near the ideal correlation line, Figure-1 A-B.

**Figure 2:**
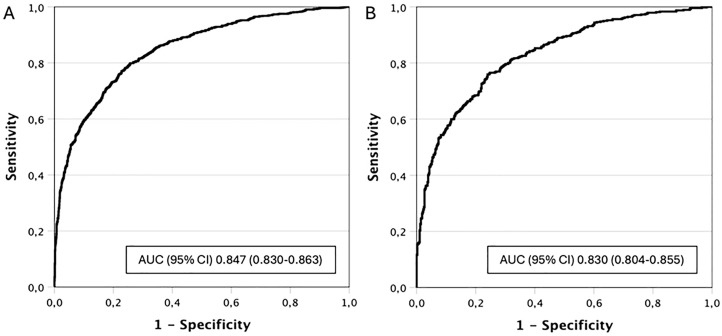
Discrimination ability of the BCN-MRI PM for csPCa detection in transrectal prostate biopsies (A) and transperineal prostate biopsies (B

ROC curves showed AUC (95% confidence intervals) of 0.847 (0.830–0.863) and 0.830 (0.804–0.855), in prostate biopsies conducted through transrectal and transperineal routes, respectively, (p =0.346), Figure-2 A-B. Specificities corresponding to 100%, 97.5%, and 95% sensitivity of the BCN-MRI PM and thresholds were analyzed. The specificity corresponding to 100% sensitivity was 1.3 and 1.8%, from the threshold of 0.36% for transrectal biopsies and 0.49% for transperineal biopsies, p = 0.438. The specificities corresponding to 97.5% sensitivity were 23.7 and 22.6%, from the threshold of 5.11% and 8.58% for transrectal and transperineal prostate biopsies respectively, p = 0395. Regarding the sensitivity of 95%, the specificities of the BCN-MRI-PM corresponded to 37.6 and 36.8%, from the threshold of 9.80 and 16.2% for transrectal and transperineal prostate biopsies respectively, p = 0387. We note that thresholds of the BCN-MRI PM were higher when the transperineal route was used. The avoided prostate biopsies with 100% sensitivity of the BCN-MRI PM were 17 (0.7%) for transrectal biopsies and 9 (0.9%) for transperineal biopsies (p =0.438). For csPCa 97.5% sensitivity, they were 344 (15%) and 112 (12.6%), respectively, (p =0.395). For csPCa 95% sensitivity, they were 557 (24.3%) and 229 (22.6%), respectively, (p =0.387).

**Figure 3:**
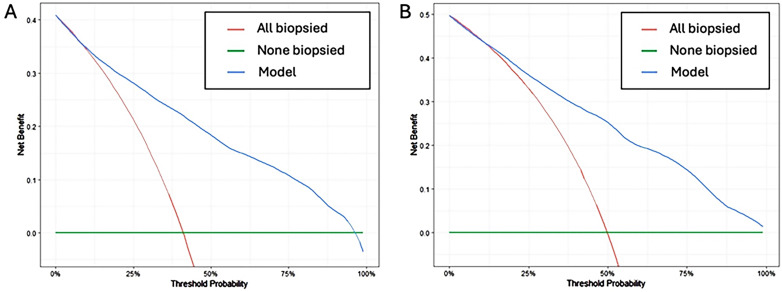
Net benefit of BCN-MRI PM application over biopsying all men through transrectal route (A) and transperineal route (B).

DCAs showed a similar net benefit of the BCN-MRI PM over biopsying all men with both prostate biopsy routes beginning at lower than 10% and 15% threshold probabilities of csPCa for transrectal and transperineal prostate biopsies, respectively, Figure-3 A-B.

## DISCUSSION

The BCN-MRI PM is based on PI-RADS score and some clinical variables that resulted independent for csPCa prediction in prostate biopsies. PSA density was the most weighed independent predictor after MRI (16, 17), that was expressed as serum PSA and MRI derived from prostate volume to avoid the manual calculation of PSA density. The BCN-MRI PM has been satisfactorily validated in men suspected of having PCa undergoing pre-biopsy mpMRI reported with the PI-RADS v2.1, and those undergoing prostate biopsies conducted via the transrectal and transperineal routes. This is important, since the BCN-MRI PM has been developed and validated using the PI-RADS v2.0 and transrectal biopsies. This successful validation guaranties the accuracy of the BCN-MRI PM predictions when PI-RADS v2.1 and transrectal biopsies are employed. The scheme of prostate biopsy conducted in this external validation study has been the same employed in the development cohort of the BCN-MRI PM, which obtained 2- to 4-core MRI-TRUS fusion-targeted biopsies of PI-RADS lesions ≥ 3 and/or 12-core systematic biopsy when the PI-RADS was <3, although some current reports suggest that systematic biopsies can be reduced (18), even to biopsy only the index lesion using a mapping scheme (19). This external validation has been successful despite the improved csPCa detection observed when the transperineal route is employed to conduct prostate biopsies. We noted that transperineal prostate biopsies detected 45.9% of csPCa while 41.7% when transrectal route was used. This finding has been previously reported, especially in anterior and apical suspicious lesions (20, 21). Similar differences in csPCa detection rates were observed in the first external validation conducted in the Barcelona metropolitan area, due to differences between the baseline characteristics of the development and validation cohorts, showing the good performance of the BCN-MRI PM (8). The risk threshold for predicting the same sensitivity for csPCa detection was higher in men who underwent transperineal prostate biopsies than in those who underwent the transrectal route. The novelty of selecting the appropriate threshold in the BCN-MRI risk calculator to select candidates for prostate biopsy is thus very useful when csPCa individualized predictions are assessed against each prostate biopsy route (8).

External validations of predictive models are necessary before employing them in new populations with different characteristics than those observed in the development cohort, and are frequently needed to perform recalibrations or adjustments of the thresholds for assessing accurate predictions (22–26). Validations are also necessary when changes in diagnostic approach occur in the same population where the predictive model was developed or in the outcome variables (27).

The European Association of Urology currently suggests the design of risk-stratified pathways using predictive models with the objective of reducing the demand for MRI exams and selecting more appropriate candidates for prostate biopsy, while also reducing the over-detection of iPCa. This is the next step in improving the current diagnostic approach for the early detection of csPCa (28). This was the reason for developing and validating the currently named BCN-predictive model 1, which is applied before the MRI exam, using the age, PCa family history, type of prostate biopsy (initial vs. repeated), DRE (normal vs. suspicious), and DRE-prostate volume category (29). Incorporation of DRE-prostate volume category was due to the importance of prostate volume as a csPCa predictive variable, since TRUS is not currently used with only this aim (30). The corresponding BCN-risk-calculator 1 is available at the same website as the BCN-MRI risk calculator, now named the BCN-risk calculator 2. Using both BCN-risk calculators, after an initial stratification based on the serum PSA level and DRE characteristic (31), we have designed a risk-organized pathway reducing MRI exams and prostate biopsies by more than a quarter with lower loss of csPCa than the currently recommended strategy of avoiding prostate biopsies in men with PI-RADS <3 (32–34). This risk-organized pathway is more efficient than that proposed by Remmers et al., based on sequential application of the Rotterdam-risk calculator 3, and the Rotterdam-MRI risk calculator (35).

Limitations of our validation study include the use of a csPCa definition in prostate biopsies which frequently results in upgrades when the entire prostate gland is analyzed. Multicentricity of the study could produce some lack of homogeneity between both series, and probably differences in quality of MRI exams (36). Additionally, inherent limitations to the predictive models developed with the binary logistic regression algorithm exist. The BCN-MRI PM, developed from a binary logistic regression, reflects the probability of csPCa based on the specific cohort characteristics and diagnostic approach at the time of its development. Changes arising in the same development population as in others where the predictive model will be applied need validations, justifying future recalibrations and adjustments of risk thresholds to ensure accurate predictions.

The real-time updating of classically developed predictive models is a current challenge (37). Dynamic training of predictive models developed with machine learning algorithms, in the setting of federated networks, has the potential to result in continuous validated risk calculators at each partner site, ensuring accurate and lasting predictions across multiple locations (38).

## CONCLUSIONS

The BCN-MRI PM has been successfully validated in men suspected of having PCa who undergo MRI exams reported with PI-RADS v2.1, and transperineal prostate biopsies. This study examined data from the csPCa early detection program of Catalonia, a region of 7.9 million inhabitants.
